# Induced expression of CCL19 promotes the anti-tumor ability of CAR-T cells by increasing their infiltration ability

**DOI:** 10.3389/fimmu.2022.958960

**Published:** 2022-08-05

**Authors:** Jian-fei Hu, Zu-wei Wang, Cheng-yu Liao, Zhi-wen Chen, Feng-ping Kang, Cai-feng Lin, Tian-sheng Lin, Long Huang, Yi-feng Tian, Shi Chen

**Affiliations:** ^1^ Shengli Clinical Medical College of Fujian Medical University, Fujian Medical University, Fuzhou, China; ^2^ Department of Hepatopancreatobiliary Surgery, Fujian Provincial Hospital, Fuzhou, China

**Keywords:** pancreatic ductal adenocarcinoma, mesothelin, nuclear factor of the activated T cell (NFAT), chemokine (C-C motif) ligand 19 (CCL19), chimeric antigen receptor-engineered T cell (CAR-T)

## Abstract

**Background:**

Chimeric antigen receptor-engineered T cell (CAR-T) therapy has shown promising potential for anti-cancer treatment. However, for pancreatic ductal adenocarcinoma (PDAC), the lack of infiltrative ability of these CAR-T cells leads to sub-optimal treatment outcome.

**Methods:**

Chemokine (C-C motif) ligand 19 (CCL19), the expression of which is regulated by the nuclear factor of activated T cell pathway, was transfected into targeting mesothelin CAR-T cells (mesoCAR-N19) using NFAT regulating element. It was expressed in activated CAR-T cells by OKT3 or mesothelin+ tumor cells but not in inactive cells. The migratory ability of these CAR-T cells was then measured. Subsequently, functional identification of these CAR-T cells was performed *in vivo*. In addition, the tumor lytic activity and proliferation of the CAR-T cells were measured *in vitro*. The degree of CAR-T cell infiltration and distribution into the PDAC tumors was examined using the immunohistochemical staining of hCD3 and the detection of CAR gene copy number by quantitative PCR. Finally, the functional assessment of chemokine (C-C motif) receptor 7 knock-out was performed in the CAR-T cells.

**Results:**

Through *in vitro* Transwell assays, it was demonstrated that mesoCAR-N19 can be specifically expressed in CAR-T cells activated by tumor cells compared with conventional mesothelin CAR-T (mesoCAR) cells. We also observed that upregulating the expression of CCL19 can increase the recruitment of additional T cells. *In vivo* studies subsequently revealed that this highly specific recruitment of T cell infiltration is associated with enhanced tumor-suppressive activities downstream.

**Conclusion:**

Induced expression of CCL19 can promote the anti-tumor ability of CAR-T cells by increasing their infiltrative ability. This study potentially uncovered novel method of activating CAR-T cells to enhance their infiltrative capacities, which offers a novel direction for PDAC treatment.

## Introduction

Pancreatic cancer (PC) is considered to be a highly aggressive malignancy that has a poor prognosis, the 5-year survival rate of which is <10% (Data collected in 2016) (1). The majority of PC cases are of the pancreatic ductal adenocarcinoma (PDAC) subtype (2). Typical treatment methods for PDAC, such as radiotherapy and chemotherapy, are unable to significantly improve patient survival (1, 2). Therefore, development of more precise and effective treatment strategies, including those of immunotherapy and adaptive immune cell therapy, is in urgent demand (3, 4).

Over the past decade, chimeric antigen receptor-modified T-cell (CAR-T) therapy has been proposed to be a potential treatment method for various malignancies such as NHL. Briefly, CAR is a fusion protein that contains an antigen-recognizing domain [single-chain variable fragment (scFv)], a hinge and transmembrane domain and several signaling domains (5). For the treatment of hematological malignancies, CAR-T has yielded highly satisfactory therapeutic effects. In diffuse large B-cell lymphoma (6, 7), the objective response rate (ORR) of CAR-T therapy can reach over 60%, whereas for multiple myeloma, the ORR of CAR-T therapy has been shown to reach over 90% (8, 9). However, in solid tumor treatment, CAR-T treatment has not been as effective (10, 11). For the treatment of hepatocellular carcinoma (12, 13), malignant glioma (14, 15) and ovarian cancer (16), CAR-T therapy has not resulted in efficacies comparable to that of leukemia. This discrepancy has been reported to be attributed to several factors. The degree of infiltration by these CAR-T cells has been observed to be poor (15, 17). In addition, the heterogeneity of the tumor tissues served as another obstacle (18, 19).The chemotaxis of CAR-T cells has not been as efficient in these solid tumors, which was inhibited further by the tumor microenvironment (17, 20–22). PC has a number of viable biomarkers with high levels of specificity, including mesothelin (23, 24) and HER2 (25, 26). However, for advanced PC, CAR-T therapy has been able to result in tumor suppression at the primary lesion due to poor infiltration and chemotaxis (16, 27).

To address this form of CAR-T cell tropism, memory T cell infiltration was increased by overexpressing the chemokine (C-C motif) ligand 19 (CCL19) in CAR-T cells (28). CCL19 is a chemokine ligand for chemokine (C-C motif) receptor 7 (CCR7), which is highly expressed on memory T cells and mature antigen-presenting cells (29). Physiologically CCL19 is expressed in the T zones in the lymph node, which enables mature fibroblasts to recruit memory T cells and mature antigen-presenting cells to activate T cells (30). Therefore, if CCL19 expression is chronically upregulated by CAR-T, then chemotaxis may become inefficient, since these CAR-T would then also home towards other tissues non-specifically instead of exclusively to the tumor tissues.

In this study, we conditionally expressed CCL19 in CAR-T cells targeting mesothelin using the nuclear factor of the activated T cell (NFAT) signaling pathway. After the CAR-T cells reach the tumor tissue and are activated by the antigen, the NFAT signaling pathway is then activated and triggers CCL19 expression. Subsequently, CCL19 expression is restricted inside the tumor tissue, which then promotes memory CAR-T infiltration into the tumor tissue.

## Materials and methods

### Cell culture

Briefly, 293T cells (cat. no. CRL-3216) were obtained from American Type Culture Collection (ATCC) and cultured with Dulbecco’s Modified Eagle’s Medium (DMEM; cat. no. 11995-065; Gibco; Thermo Fisher Scientific, Inc.) supplemented with 10% fetal bovine serum (FBS; cat. no. 10099-141; Gibco; Thermo Fisher Scientific, Inc.). BXPC-3 (CRL-1687, ATCC) and AsPC-1 (CRL-1682, ATCC) cells were obtained from ATCC and cultured in RPMI-1640 medium (cat. no. 11875093; Gibco; Thermo Fisher Scientific, Inc.) supplemented with 10% FBS. Human T cells were obtained from Allcells (cat. no. PB009-CD3-F). Both of the cells were cultured in 5% CO_2_ at 37°C.

### Lentivirus preparation

**Figure 1:**
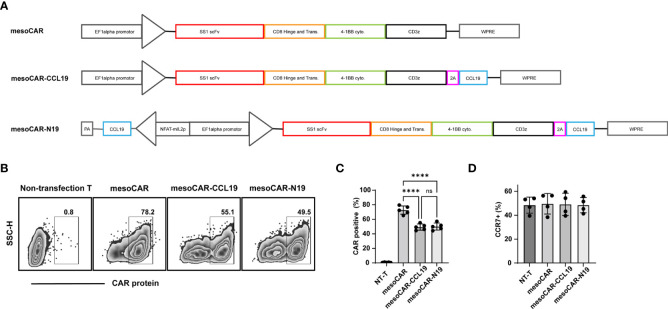
Characterization of chimeric antigen receptor T (CAR-T) cells: **(A)** Schematic diagram of the CAR structure; **(B)** Detection of CAR protein transfection efficiency by flow cytometry and **(C)** statistics performed on five donor-derived cells; **(D)** Detection of C-C motif chemokine receptor 7 positivity by flow cytometry. Error bars represent the mean ± standard deviation (n = 5). ^****^P<0.0001. ns, no significant.

The CAR and its promotor were designed as shown in **Figure 1A**. The genes and their promoters were fully synthesized and inserted into the lentiviral plasmid vector pLVX-IRES-GFP. For lentivirus particle packaging, this core lentiviral plasmid and two helper plasmids, pMD2.G and psPAX-2 were co-transfected into 293T cells using Lipofectamine 3000 (cat. no. L3000008;ThermoFisher Scientific, Inc.) transfection reagent. After 48 h, the supernatant was harvested and the lentivirus was concentrated according to the protocols of Lenti-X Concentrator (cat. no. 631232; Takara Bio, Inc.).

### CAR-T cell preparation

Human T-cells were activated by the Dynabeads™ Human T-Activator CD3/CD28 (cat. no. 11161D; Thermo Fisher Scientific, Inc.) and cultured for 24 h. Subsequently, the lentivirus was added with a multiplicity of infection of 2. The cells were cultured with X-VIVO-15 (Lonza Group, Ltd.) with 100 IU/ml interleukin (IL)-2 (cat. no. 200-02; PeproTech China). The medium was changed every 2 days and the cells were cultured for 10 days after activation.

### ELISA

To stimulate mesothelin CAR-T (mesoCAR) cells into expressing CCL19 in mesoCAR-N19 cells, we activated mesoCAR-N19 CAR-T cells using PHA (sigma, 11249738001) or tumor cells. The PHA-L concentration was kept at a 500-fold dilution, whereas the ratio of CAR-T cells to tumor cells in the co-culture was 1:1. The co-culture stimulation time with tumor cells or PHA-L was 6 h at 37°C. The cell supernatant was then collected afterwards and assayed using the Human CCL19/MIP-3βDoust ELISA (cat. no. DY361; R&D systems, Inc.) according to the manufacturer’s protocols. For the mouse plasma samples, blood samples were collected through the eye socket and centrifuged at 500 ×g for 10 min at room temperature, after which the supernatant was collected and assayed using the Human CCL19/MIP-3βDuoSet ELISA according to the manufacturers’ protocols.

### Flow cytometry

In total, 1×10^6^ cells were placed in a tube and centrifuged at 500 ×g for 5 min at room temperature, before the supernatant was discarded. The cells were then resuspended with phosphate-buffered saline (PBS) containing 2% bovine serum albumin, after which 1 μg antibody was added to the tube, followed by incubation for 30 min at 4˚C. To this solution, 1 ml PBS solution was added before the mixture was centrifuged at 500 ×g for 5 min at room temperature. The supernatant was then discarded and the cells were resuspended with 200 μl PBS before flow cytometry (CantoII, BD). The antibodies used were as follows: CCR7 (cat. no. 557734; BD Biosciences), CCL19 (cat. no. 566523; BD Biosciences), mesothelin (cat. no. 530203; Biolegend, Inc.), streptavidin (cat. no. 554067; BD Biosciences) and protein L (cat. no. RPL-P81Q7).

### Cytotoxicity

CAR-T cells and target or non-target cells were first mixed in a graded ratio, whilst equivalent densities of either CAR-T cells alone or tumor cells alone were used as corresponding controls. After co-culturing for 4 h at 37°C, the 96-well plates were centrifuged at 500 ×g for 5 min at room temperature before 50 μl of this supernatant solution was subjected to lactate dehydrogenase quantification using the CytoTox 96^®^ Non-Radioactive Cytotoxicity Assay (cat. no. G1780; Promega Corporation). After reading the optical density value at 490 nm using a microplate reader, the cytotoxicity was calculated according to the following equation: Cytotoxicity (%) = (Experimental group - T-cell only - tumor only-3 × medium control)/tumor_max_-tumor_auto_.

### Transwell assays

We used Transwell plates (cat. no. 3414; Corning, Inc.) for the present study. Carboxyfluoresceinsuccinimidyl ester (CFSE)-labeled or unlabeled CAR-T cells were added to the upper chamber (1×10^5^ cells/well) in RPMI 1640 medium. By contrast, the lower chamber contained pre-coated immobilized OKT3 (5 μg/ml) or AsPC-1 cells (1×10^5^ cells) in RPMI 1640 medium supplement 2% FBS. The concentration of CCL19 was 10 ng/ml.

### Cytokine assay

For the detection of cytokines released after CAR-T cell co-culturing with AsPC-1 cells, we mixed CAR-T cells with BxPC-3 or AsPC-1 cells for 18 h in RPMI 1640 medium supplement 2% FBS. After this, the supernatant was collected before six types of cytokines were tested using the Human Th1/Th2 Cytometric Bead Array (CBA) Kit (cat. no. 551809; BD Biosciences). Cytokine detection were performed by flow cytometry (BD FACSCanto™ II; BD Biosciences) and analyzed by the Flowjo software.

### CFSE assay

The cells were labeled with the CellTrace™ CFSE Cell Proliferation Kit (cat. no. 65-0850-84; ThermoFisher Scientific, Inc.) according to the manufacturer’s protocols. After labeling, the CAR-T cells were co-cultured with Aspc-1 tumor cells at a 1:1 ratio for 2 days at 37°C, followed by staining of the cells with an anti-human CD3 antibody (Biolegend, 300311) and analyses by Flow cytometry.

### Cas9/CRISPR gene editing

The T cells were activated and transduced with lentivirus as previously described. In total, 2×10^6^ CAR-T cells were electroporated, 50pM CAS9 protein (Novoprotein, E365) and 300 pM single guide (sg) RNA were incubated together for 10 min at 25˚C to obtain Cas9/sgRNA ribonucleoprotein. CAR-T cells were resuspended in 20 μlP3 Buffer(Lonza Group, Ltd.), combined with RNP and electroporated using a 4D-Nucleofector™(Lonza Group, Ltd.) with pulse code E0115. Next, CAR-T cells were cultured with T cell medium as previously described for 4-5 days. The sgRNA targeting sequence in the CCR7 gene was 5′-CGCAACTTTGAGCGCAACA-3’ (CRISPRD HSPD0000007879).

### Mouse experiments

Nod Scidγ mice aged 6 weeks were purchased from Shanghai Model Organisms Center, Inc, All mice were female. To obtain the pancreatic tumor xenograft model, 2×10^6^AsPC-1 cells in 100 μl PBS were injected into the right flank of mice with Matrigel (Corning, Inc.). When the mean tumor volumes reached 300 mm^3^, we injected 2×10^6^ CAR-T or 5×10^5^ CAR-T cells suspended in 200 μl PBS, into the mice by tail vein injection before changes in tumor volume were observed every 7 days. The tumor volumes were monitored by caliper measurement and the volumes were calculated as follows: Volume = (length × width^2^)/2.

For *in vivo* imaging experiments, 2×10^6^ AsPC-1-lucferase cells suspended in100 μl PBS were injected into the right flank of mice with Matrigel (Corning, Inc.).After CAR-T treatment, the images of mice were captured using IVIS^®^Lumina SeriesIII (PerkinElmer, Inc.) after luciferin (*In vivo* grade) treatment. The mice were sacrificed with an overdose of 10% pentobarbital sodium (100 mg/kg; intraperitoneal injection) and death was confirmed by the disappearance of heartbeat. The mouse experiments were approved by the Animal Research and Care Committee of Fujian Provincial Hospital, Shengli Clinical Medical College of Fujian Medical University (Fuzhou, China) and performed in accordance with the NIH guidelines for Laboratory Animals and established Institutional Animal Use and Care protocols at the Fujian Provincial Hospital, Shengli Clinical Medical College of Fujian Medical University.

### IHC staining and quantitative PCR

Immunohistochemistry was performed on paraformaldehyde-fixed and paraffin-embedded samples. The tumors were excised using a microtome and stained according to standard procedures. The sections were stained with an anti-human CD3 antibody (cat. no. ab16669; Abcam) and then with HRP-conjugated Goat Anti-Rabbit IgG (cat. no. ab6721; Abcam).

For qPCR, the genomic DNA was extracted by using the TIANamp Genomic DNA Kit (cat. no. DP304; Tiangen Biotech Co., Ltd.). qPCR assay was performed according to standard procedures. The primer and probe sequences were as follows: Forward, 5’- CTGGCTGCAGTACGTGATTC-3’ and reverse, 5’-GGCCTCGAACTCTCCCACC-3’ and probe, 5’ fluorescein amidites-GATCCCGAGCTTCGGGTTGGAAGT-TAMRA 3’.

### Statistical analysis

The statistical analysis was performed with using the GraphPad Prism software 9.2 (GraphPad Software, Inc.). One-way ANOVA and two-way ANOVA with Bonferroni *post hoc* test or t-tests unpaired were performed for different conditions. Statistical significance was represented by the following P-values: ^****^P<0.0001,^***^P<0.001, ^**^P<0.01 and ^*^P<0.05 or no statistical significance (ns).

## Results

### CAR-T preparation and phenotype

The lentiviral structures are shown in **Figure 1A**. In brief, the targeting mesothelin CAR (mesoCAR) construct was generated using a tandem construct encoding SS1 ScFv domain that was fused using the CD8 hinge and the 4-1BB and CD3ζ intracellular signaling regions. In addition, the co-expression system of CCL19 and mesoCAR was created by fusing the mesoCAR with human CCL19 cDNA using a 2A peptide to produce the mesoCAR-CCL19 construct. The conditional NFAT signaling-inducible expression of CCL19 was controlled by an NFAT-binding motif linked to a minimal IL-2 promoter (mesoCAR-N19). The expression of CAR in these CAR-T cells is shown in **Figures 1B, C**. When compared with that in mesoCAR, the transfection efficiency of CAR in mesoCAR-CCL19 and mesoCAR-N19 cells was significantly lower. This difference may have been caused by the larger gene insertions that took place in themesoCAR-CCL19 and mesoCAR-N19 constructs. However, there was no memory phenotypic difference among the three CAR-T cell subtypes (**Figure 1D**).

### CCL19 expression

**Figure 2:**
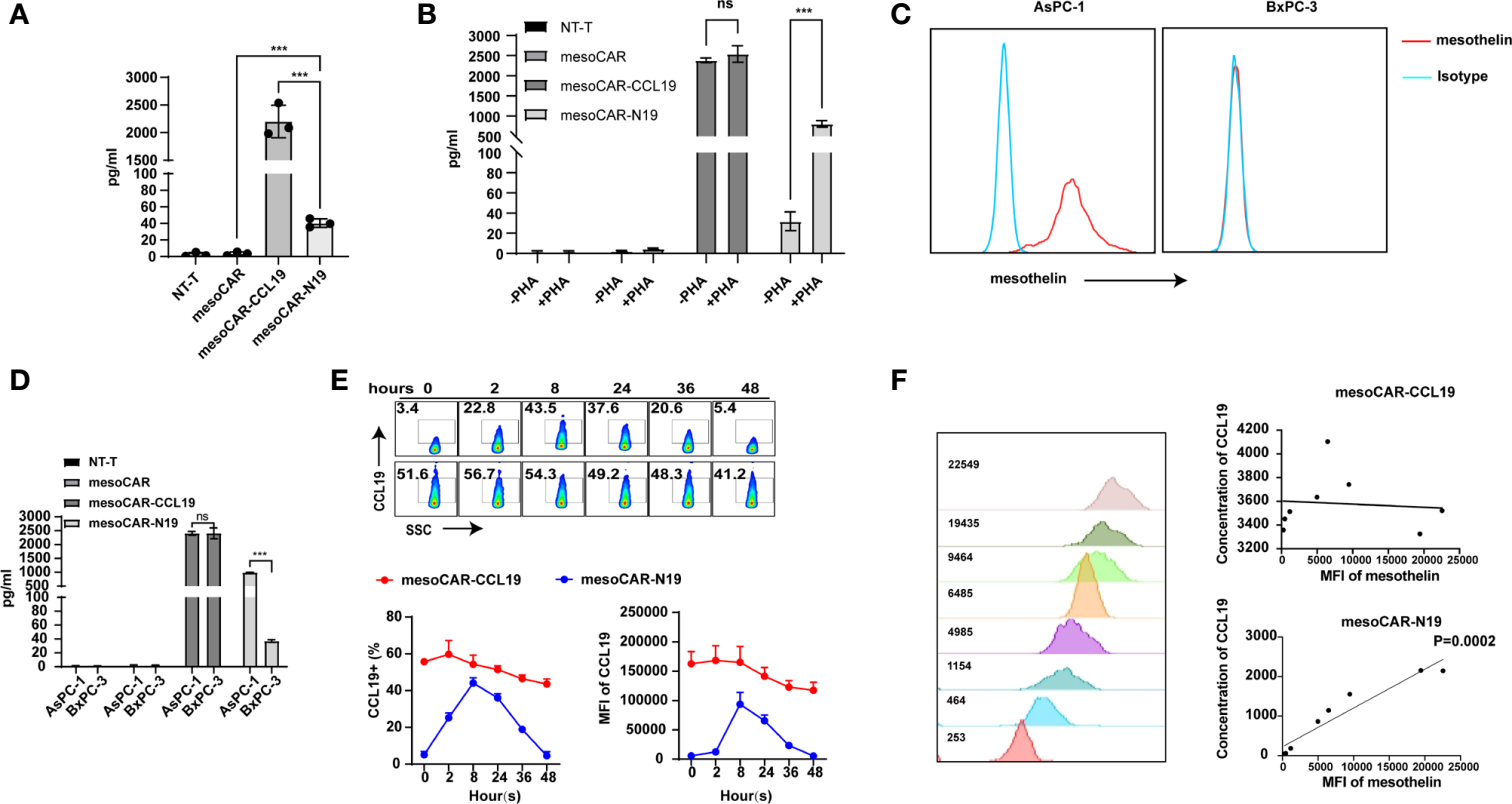
Identification of C-C motif chemokine ligand 19 (CCL19) expression. **(A)** Resting CCL19 secretion level measured by ELISA; **(B)** Detection of CCL19 secretion in chimeric antigen receptor T (CAR-T) cells before and after activation with PHA-L by ELISA; **(C)** Detection of mesothelin expression in the cancer cell lines; **(D)** Detection of CCL19 expression in CAR-T cells after co-culturing with AsPC-1 cells by ELISA; **(E)** Expression of CCL19 at different time points after co-culturing with AsPC-1 cells as detected by flow cytometry; **(F)** Correlation analysis of mesothelin expression and CCL19 secretion. Error bars represent the mean ± standard deviation (n = 6). ^***^P<0.001; ns, no significance.

Next, we detected CCL19 levels in the supernatant. As shown in **Figure 2A**, CCL19 secretion could not be detected in the untransfected (NT-T) or mesoCAR cells. However, unlike mesoCAR-CCL19 cells, which showed markedly increased CCL19 secretion, CCL19 could only be weakly detected in the supernatant of mesoCAR-N19 cells. This difference could be attributed to the hypothesis that the NFAT promoter is inactive. Therefore, we measured the levels of CCL19 secreted by the CAR-T cells with and without PHA-L treatment for 6 h. As shown in **Figure 2B**, CCL19 secretion was significantly higher in the mesoCAR-N19 group stimulated with PHA compared with those in mesoCAR-N19 not stimulated with PHA. By contrast, CCL19 secretion in the mesoCAR-CCL19 cells was not significantly different between the PHA-treated and untreated groups. Subsequently, we investigated whether CCL19 expression in the mesoCAR-N19 cells can be induced by mesothelin-positive or negative tumor cells. Mesothelin expression levels were measured using flow cytometry (**Figure 2C**). Mesothelin expression was detected in AsPC-1 cells at high levels but not in BxPC-3. After co-culturing with AsPC-1 or BxPC-3 for 6 h, we collected the culture supernatant before measuring the CCL19 content using ELISA (**Figure 2D**). In the mesoCAR-N19 cells, CCL19 secretion could be induced by AsPC-1 but not by BxPC-3 cells. To investigate the CAR-T CCL19secretion kinetics, we next co-cultured the CAR-T cells with mesothelin-positive AsPC-1cells and sampled them for CCL19 secretion at multiple time points using flow cytometric analysis. As shown in **Figure 2E**, CCL19 secretion started at 2 h after co-culturing, before the highest levels of secretion were maintained for 24 h and gradually declining after 24 h. The decrease may have been due to the complete clearance of the tumor cells. By contrast, mesoCAR-CCL19 cells consistently released high levels of CCL19, which only decreased slightly after 24 h. This was most likely due to the deteriorating culture conditions. To investigate the correlation between mesothelin expression and CCL19 secretion, we constructed eight cell lines with differential mesothelin expression levels based on the BxPC-3 cell line. CAR-T cells were then co-cultured with these tumor cells for 16 h, before the concentration of CCL19 secreted into the supernatant was detected. In the mesoCAR-N19 group, CCL19 secretion showed a positive correlation with the levels of mesothelin expression, but not mesoCAR-CCL19 group (**Figure 2F**). Altogether, these results suggest that NFAT signaling-regulated CCL19 can be induced by tumor cell-surface mesothelin *in vitro*.

### Chemotaxis of CCL19

**Figure 3:**
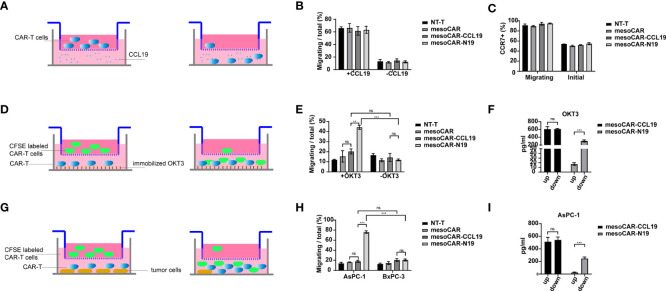
Migratory ability of the chimeric antigen receptor T (CAR-T) cells. **(A)** Schematic diagram of the experimental principle; **(B)** CAR-T cell migration and quantification; **(C)** C-C motif chemokine receptor 7 expression on cells undergoing migration detected by flow cytometry; **(D)** Schematic diagram of the experimental principle; **(E)** CAR-T cell migration and quantification; **(F)** Detection of C-C motif chemokine ligand 19 (CCL19) concentration in the upper and lower chambers by ELISA; **(G)** Schematic diagram of the experimental principle; **(H)** CAR-T cell migration and quantification; **(I)** Detection of CCL19 concentration in the upper and lower chambers by ELISA. Migration efficiency was calculated as cells undergoing migration/total number of cells in the upper chamber. Error bars represent the mean ± standard deviation (n = 6). ^**^P<0.01; ^***^P<0.001; ns, no significance.

CCR7 is a known receptor for CCL19 that is expressed on the surfaces of memory T cells and can mediate T cell chemotaxis toward areas of high CCL19 concentrations. Therefore, to assess ifCCL19 can recruit memory CAR-T cells, we performed a Transwell assay. The experimental schematic is shown in **Figure 3A**. In a 6.5-µm Transwell plate, serum-free RPMI 1640 culture medium containing a gradient concentration of CCL19 was added to the lower chamber, whereas CAR-T cells suspended in serum-free RPMI1640 media were added into the upper chamber. After 4 h, cells in the lower chamber were counted and collected for flow cytometry analysis. The results are presented in **Figures 3B, C**. There was no significant difference in the number of migratory cells in the lower chamber, since the proportion of the memory T cell population was similar in the groups. The CCR7 expression-positivity rate of the recruited cells in the lower chamber was significantly higher compared with that of the pre-experimental period in all the groups. Next, we assessed whether CCL19 secreted by the CAR-T cells serves a recruitment function. Since the mesoCAR-N19 cells can only release CCL19 in the activated state, we constructed two activation models, namely the coated OKT3 antibody model and the tumor cell stimulation model. The experimental models are shown in **Figures 3D, G**. A total of 8 h before the start of the experiment, we replaced the CAR-T cell culture medium with a RPMI 1640 medium without 2% FBS to avoid residual CCL19. The CAR-T cells to be added to the upper chambers were then stained with CFSE before the lower chamber was treated with coated OKT3 antibodies or mesothelin-positive tumor cells. An equivalent number of unlabeled CAR-T cells were added directly to the lower chamber whereas an equivalent number of CFSE-labeled CAR-T cells were added to the upper chamber. In total, 6 h later, cells from the lower chamber were collected for flow cytometry analysis and the number of CFSE-positive cells was counted. The results are presented in **Figures 3E, H**. MesoCAR-N19 cells demonstrated a potent chemoattractant ability compared with control group and mesoCAR-CCL19 group in both models tested, namely with OKT3 antibodies and with mesothelin-positive tumor cells, whilst cells in the other groups did not show significant recruitment ability relative to the inactivated state. In particular, mesoCAR-CCL19 cells, which chronically express CCL19, did not increase recruitment capacity. This may be due to the absence of a CCL19 gradient between the upper and lower chambers. Therefore, we collected the supernatants from the upper and lower chambers for ELISA, the results of which are depicted in **Figures 3F, I**. Only mesoCAR-N19 cells demonstrated a CCL19 concentration difference. In the mesoCAR-CCL19 group, despite the higher CCL19 concentrations, there was no significant difference in the concentrations between the upper and lower chambers. These findings suggest that mesoCAR-N19 cells can effectively release CCL19 in sites of activation to recruit other T cells.

### *In vivo* tumor suppression

**Figure 4:**
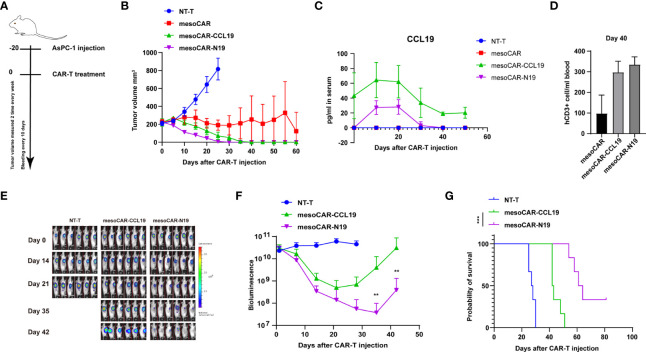
Analysis of chimeric antigen receptor T (CAR-T) cell function *in vivo*. **(A)** Schematic diagram of the experimental design; **(B)**Tumor volume measurements; **(C)** Changes in C-C motif chemokine ligand 19 levels in the mouse plasma as measured using ELISA; **(D)** Detection of CD3-positve cells using flow cytometry on day 40. **(E)** Images of tumors after CAR-T treatment; **(F)** Bioluminescence images and luminescence curve; **(G)** Mouse survival presented as Kaplan-Meier curves. In **(B, C)**, the error bars represent the mean ± SD whereas in **(F)** the error bars represent the means± standard error of the mean (n =6). **p<0.01

To evaluate the potential tumor-suppressive efficiency of mesoCAR-N19 cells, we used the AsPC-1 cell line xenograft mouse model (**Figure 4A**). Changes in tumor volume were shown in **Figure 4B**. We found that the tumor suppression efficiency of mesoCAR-N19 cells was significantly higher compared with that of mesoCAR and mesoCAR-CCL19 cells in the initial stage (**Figure 4B**). The plasma CCL19 profile in the mice is shown in **Figure 4C**. Significantly higher levels of CCL19 secretion could be detected in both the mesoCAR-CCL19 and mesoCAR-N19 groups but not in the control or mesoCAR groups. CCL19 secretion in the mesoCAR-CCL19 group was significantly higher compared with that in the mesoCAR-N19 group, which maybe because mesoCAR-N19 tended to be only activated in the tumor tissue site. After tumor recession, traceable quantities of CCL19 expression could be detected in serum in the mesoCAR-CCL19 group but not in the mesoCAR-N19 group, although the presence of CD3-positive T cells could be detected in peripheral blood by flow cytometry in both of these CAR-T groups (**Figure 4D**). In addition, in peripheral blood, the CD3-positivity rates in the mesoCAR-CCL19 and mesoCAR-N19 groups were significantly higher compared with that in the mesoCAR group. This may be caused by the mesoCAR-CCL19 and mesoCAR-N19 cells exhibiting superior CAR-T cell recruitment capabilities compared with those of mesoCAR cells due to the expression of CCL19. As a result, after these CAR-T cells were activated and proliferate, the number of CAR-T cells in the bloodstream would be higher compared with that of mesoCAR. To further compare the tumor suppressive effects of mesoCAR-N19 and mesoCAR-CCL19 CAR-T cells *in vivo*, we reduced the number of CAR-T cells injected to 5×10^5^. MesoCAR-N19 cells showed significantly more potentoncolytic effects compared with those by mesoCAR-CCL19 cells (**Figures 4E, F**). In addition, the mesoCAR-N19 cells significantly prolonged the survival of mice compared with that in mice injected with NT-T and mesoCAR-CCL19 cells (**Figure 4G**). These results suggest that mesoCAR-N19 cells confer superior tumor suppressive effects compared with conventional CARs.

### Assessment of the impact of CCL19 on CAR-T function

**Figure 5:**
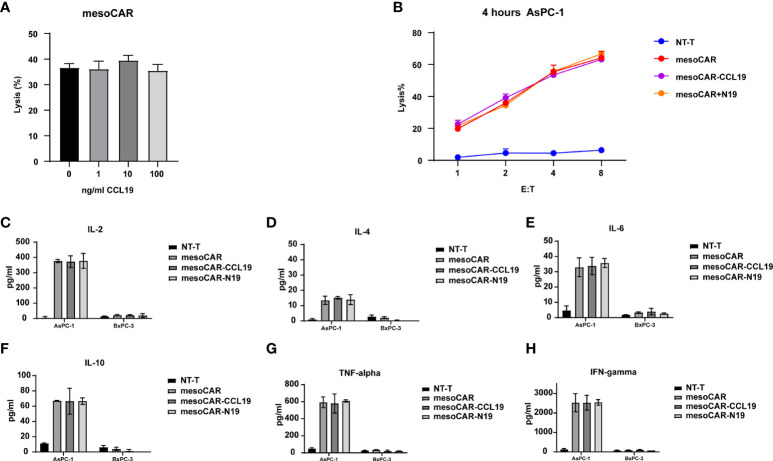
Chimeric antigen receptor T (CAR-T) cell activity assays *in vitro*. **(A)** Killing activity assay of the mesothelin CAR-T cells with different concentrations of C-C motif chemokine ligand 19 on AsPC-1 cells by LDH assay; **(B)** Killing activity of CAR-T cells on theAsPC-1 cell line without CCL19; Secretion of **(C)** interleukin (IL)-2, **(D)** IL-4, **(E)** IL-6, **(F)** IL-10, **(G)** tumor necrosis factor-α and **(H)** interferon-γafter co-culturing the CAR-T cells with tumor cells, respectively (n = 3).

**Figure 6:**
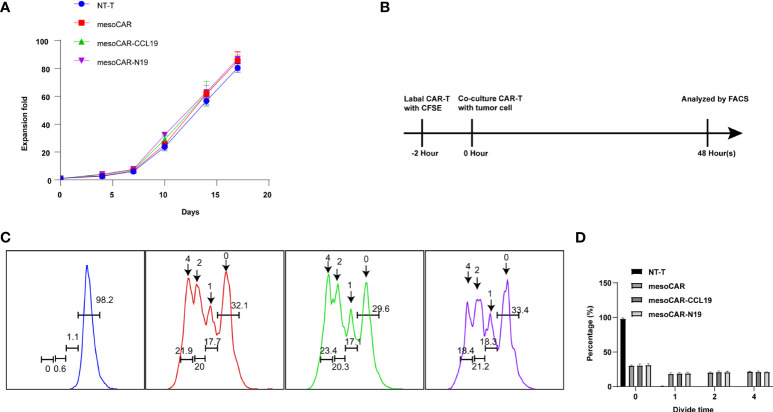
Chimeric antigen receptor T (CAR-T) cell proliferation *in vitro*. **(A)** The proliferation curve of CAR-T cells in *in vitro* culture by cell count; **(B)** Schematic diagram of the principle of carboxyfluoresceinsuccinimidyl amino ester(CFSE) staining; **(C)** Flow cytometry detection of CFSE fluorescence; **(D)** Cell division generation statistics summarized from CFSE staining results. n = 6.

*In vivo* experiments have shown that CAR-T expressing CCL19 can consistently and conditionally exert beneficial tumor-suppressive effects. Therefore, we next attempted to investigate if CCL19 is able to regulate the physiology of CAR-T itself. We assessed the killing activity of mesoCAR cells in the presence or absence of CCL19 at a final concentration of 50 ng/ml against mesothelin-positive cell lines. The presence of CCL19 in mesoCAR cells did not affect their mesothelin-positive tumor cell Aspc-1 killing activity over 4 h (**Figure 5A**). Subsequently, we tested the killing activity of the three CAR-TS against AsPC-1 cells over a 4-h period. There was no significant difference among the three CAR-T groups without CCL19 supplement (**Figure 5B**). In addition, cytokine release was measured. The type and quantity of cytokine released from each group of CAR-T cells are shown in **Figures 5C–H**. There was no significant difference in the effects of CCL19 on three group CAR-T cells. In addition, CCL19 did not significantly enhance the proliferative ability of the CAR-T cells that did not encounter the tumor cells, where there was no significant difference in the proliferation rate among the groups (**Figure 6A**). We next labeled CAR-T cells with CFSE before co-culturing them with mesothelin-positive tumor cells for 2 days to estimate the rate of CAR-T cell division (**Figure 6B**). The results showed that neither the addition of CCL19 nor the overexpression of CCL19 could increase the rate of T cell division (**Figures 6C, D**). These results suggest that CCL19 itself cannot increase the killing activity of CAR-T on the tumor cells. Therefore, the potent tumor-suppressive effects of CAR-T cells expressing CCL19 are unlikely to be directly derived from CCL19.

### Effect of CCL19 on the distribution of T cells *in vivo*

**Figure 7:**
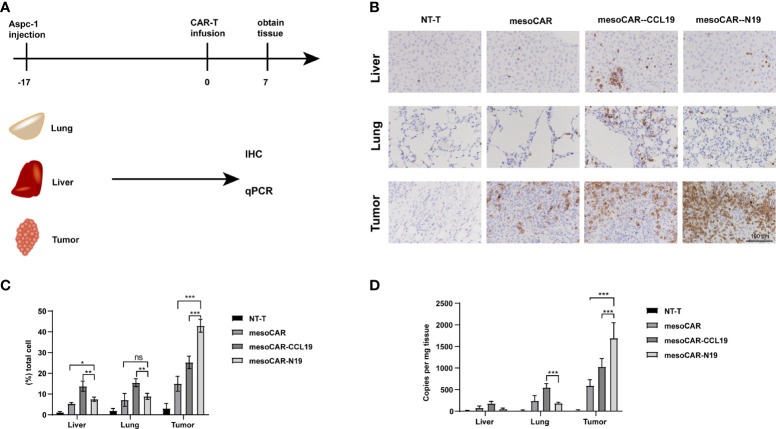
Chimeric antigen receptor (CAR) T (CAR-T) cell infiltration and distribution. **(A)** Schematic diagram of experimental design; **(B)** Immunohistochemical staining of hCD3 and **(C)** quantification; **(D)** Detection of CAR gene copy numbers by quantitative PCR. Error bars represent the mean ± standard deviation (n = 6).^*^P<0.05; ^**^P<0.01; ^***^P<0.001; ns, no significance.

The AsPC-1 tumor xenograft mouse model was used to assess the distribution of T cells. When the tumor volume reached ~300 mm^3^, we injected the CAR-T cells through the tail vein. Days later, we extracted the mouse liver, lung and tumor tissues to determine their mass before dividing them into two groups. One group was used for the immunohistochemical study of CD3 cell infiltration, whereas the other was used for analyzing the copy number of the CAR gene after genome extraction (**Figure 6A**). Both groups of CAR-T cells expressing CCL19 showed rapid infiltration in the tumor tissues. Specifically, mesoCAR-N19 cells exhibited significantly higher degrees of tumor infiltration compared with those in the other groups. By contrast, in the lung and liver tissues, mesoCAR exhibited higher levels of residency. MesoCAR-N19 also demonstrated a greater ability to specifically infiltrate the tumor tissues compared with that by mesoCAR-CCL19 cells (**Figures 7A–D**).

**Figure 8:**
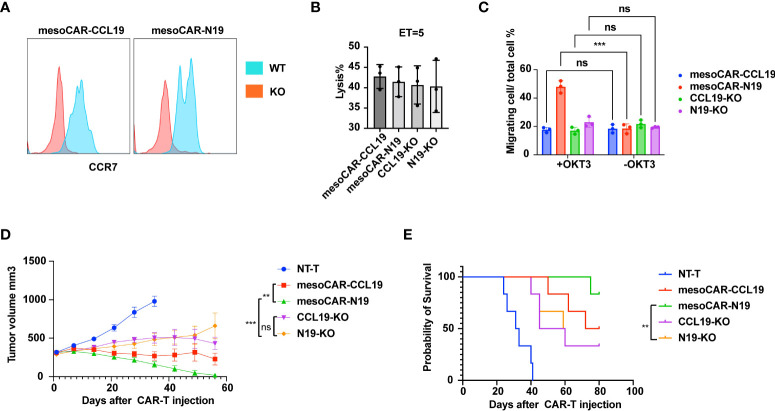
Functional assessment of C-C motif chemokine receptor 7 (CCR7) knockout in chimeric antigen receptor T (CAR-T) cells. **(A)** CCR7 expressing detection on surface of wild type or CCR7 knockout CAR-T cells; **(B)** Tumor cell lysis assessment *in vitro* at 5 ET ratio(n = 6); **(C)** Migratory capacity estimation using Transwell assays (n = 6); **(D)** Tumor suppression curve; **(E)** Mouse survival presented as Kaplan-Meier curves (n =6). Error bars represent the mean ± standard deviation (n = 6). ^**^P<0.01;^***^P<0.001; ns, no significance.

To verify that the stronger tumor suppressive effects of mesoCAR-N19 cells was mediated through enhanced tumor homing, the CCR7 expression was knocked down in the CAR-T cells by Cas9/CRISPR before the CAR^+^CCR7^-^ cells were sorted (CCL19-KO and N19-KO). CCR7 expression was first measured by flow cytometry (**Figure 8A**). After the CCR7 expression was knocked out, it could not be detected in cell surface by flow cytometry. The killing activity by CAR-T cells (mesoCAR-CCL19, mesoCAR-N19, CCL19-KO and N19-KO) of AsPC-1 cells was assessed. As **Figure 8B** shows, the capacity of cell killing was not significantly different among the CAR-T cell groups. After CCR7expression was knocked out, the migratory capacity of the CAR-T cells (mesoCAR-CCL19, mesoCAR-N19, CCL19-KO and N19-KO) were evaluated (**Figure 3E**). As **Figure 8C** shows, activated N19-KO cells by OKT3 in the lower chamber could not improve the migration rate of CAR-T cells in the upper chamber.

Furthermore, *in vivo* tumor experiments were performed. Briefly, AsPC-1 cells were injected subcutaneously. After the mean tumor volume reached >300 mm^3^, 5×10^5^ CAR-T cells were injected intravenously and tumor volume was measured once a week. As **Figure 8D** shows, the tumor suppressive activity of N19-KO cells was significantly weaker compared with that of mesoCAR-N19 cells but had no significant difference with that of CCL19-KO cells. These observations suggest that mesoCAR-N19 cells can attract additional CAR-T cells to the tumor to facilitate suppression by enhancing infiltration into the tumor tissues.

## Discussion

In this study, we designed a CAR-T targeting mesothelinco-expressing CCL19 downstream of an inducible expression system regulated by NFAT signaling. Furthermore, we compared the infiltrative capabilities and killing abilities of these CAR-T cells with those of the mesoCAR-T cells without CCL19 expression in addition to those of chronically CCL19-expressing CAR-T cells as controls. Both types of CAR-Ts overexpressing CCL19 (mesoCAR-CCL9 and mesoCAR-N19) exhibited higher tumor infiltration abilities when compared with those by conventional CAR-T (mesoCAR-T). However, mesoCAR-N19 cells had higher tumor-specific infiltration ability and superior killing effects on the tumor compared with those by mesoCAR-CCL9 cells.

For the treatment of solid tumors with CAR-T cell therapy, the level of CAR-T cell infiltration is critical for determining the therapeutic outcome (10, 17). However, CAR-T infiltration into solid tumors has been poor owing to the lack of effective chemokines for inducing CAR-T tropism in the tumor tissues (31). Inducing the expression of chemokines in CAR-T cells to promote additional CAR-T cell infiltration has been proposed to be a feasible approach. It has been previously shown that overexpressing CCL19 in CAR-T cells can promote the infiltration of CAR-T cells and other immune cell types such as dendritic cell to enhance therapeutic effects (28, 32). However, at the initial stages following CAR-T cell transfusion, the destination of CAR-T cells is not tumor-specific. In particular, after intravenous administration, CAR-T cells were found to preferentially enter the pulmonary circulation before homing to various secondary lymph nodes throughout the body (33). Therefore, if the CAR-T cells were programmed to chronically overexpress CCL19, it would not be able to efficiently recruit other CAR-T cells or immune cells to specifically infiltrate the tumor tissue. Therefore, use of an inducible signaling pathway, namely the NFAT signaling pathway which is necessary signaling for T cell activated, to regulate CCL19 expression was attempted for the present study. The rationale is that only CAR-T cells that have successfully infiltrated into the tumor tissues can be activated by the tumor cells and release CCL19 to recruit other immune cells for infiltration. A similar experimental design has been previously applied to CAR-T cells expressing IL-12 (34). Since mesothelin is also weakly expressed in the normal tissues, such as the peritoneum and pericardium, off-target toxicity has been observed in previous clinical studies testing mesothelin CAR-T cells (35). If CAR-T or other immune cells can be specifically recruited to the tumor tissues, then this off-target toxicity can theoretically be alleviated.

CCL19 is mainly expressed in cells in the lymph node and is used to recruit memory T cells and mature antigen-presenting cells. In terms of CAR-T cells, it has been proposed that cells with a memory phenotype tend to have a high proliferative capacities and resistance to depletion *in vivo*. Therefore, there is frequently an association between the size of the memory cell population and beneficial therapeutic effects in hematoma treatment (36). In the case of solid tumors, since memory CAR-T cells express CCR7, they may tend to home to lymph nodes but fail to infiltrate into the tumor tissue *in vivo*. Therefore, in the present study, conditionally-expressed CCL19 could facilitate the recruitment of CAR-T cells. In particular, memory CAR-T cells may infiltrate into sites with high CCL19 concentration to augment the suppressive effects in the tumor.

In addition, another factor limiting the use of CAR-T cells in the clinic is the time required for CAR-T cell preparation. This process typically takes 17 days, which is dominated by the expansion of CAR-T cells required to obtain clinically sufficient doses (37). For solid tumors, the general dose of CAR-T cells needs to be higher compared with that for lymphomas, which is mainly due to insufficient CAR-T infiltration. In the present study, inducing the expression of CCL19 effectively directed the chemotactic CAR-T cells to specifically infiltrate the PC tumor tissue. This infiltration can potentially lower the CAR-T dose required in the clinical setting. The reduction in the preparation cost may also improve the therapeutic effects because of the shortened time period required for CAR-T cell division and differentiation.

In summary, the NFAT-regulated expression of CCL19 in mesothelin-targeting CAR-T cells in the present study was able to effectively lyse the PC tumor cells. Furthermore, these cells can precisely release CCL19 in the tumor tissues to recruit additional memory T cells, including memory CAR-T cells, to infiltrate inside the PC tumor tissues for enhancing the tumor suppressive effects.

## Data availability statement

The original contributions presented in the study are included in the article/supplementary material. Further inquiries can be directed to the corresponding author/s.

## Ethics statement

The animal study was reviewed and approved by Ethics committee of Fujian Provincial Hospital.

## Author contributions

Conception and design: LH, YT and SC. Experiments: JH, ZW and CyL. Acquisition and analysis of data: JH, ZW, CyL, ZC, FK and CfL. Interpretation of data: TL and LH. All authors contributed to the article and approved the submitted version.

## Funding

The present study was supported by the National Natural Science Foundation of China (#82173250 to SC), Joint Funds for the innovation of science and technology, Fujian Province (#2018Y9098 to SC), High-level hospital foster grants from Fujian Provincial Hospital (#2019HSJJ13 to SC), Education and Scientific Research Foundation of Fujian Province (#2060402 to SC), Fujian Provincial Health and Family Planning Research Medical Inovation Project (#2019-cx-3 to SC), Fujian Medical Innovation Project (#2020CXA001 to YT) and Fujian Natural Science Foundation (#2020J011097 to YT), Fujian Province Health and Middle-aged and Young Scientific Research Major Project (#2021ZQNZD001 TOSC).

## Conflict of interest

The authors declare that the research was conducted in the absence of any commercial or financial relationships that could be construed as a potential conflict of interest.

## Publisher’s note

All claims expressed in this article are solely those of the authors and do not necessarily represent those of their affiliated organizations, or those of the publisher, the editors and the reviewers. Any product that may be evaluated in this article, or claim that may be made by its manufacturer, is not guaranteed or endorsed by the publisher.

## References

[1] Ansari D Tingstedt B Andersson B Holmquist F Sturesson C Williamsson C. Pancreatic cancer: yesterday, today and tomorrow. Future Oncol (2016) 12:1929–46. doi: 10.2217/fon-2016-0010 27246628

[2] Goral V. Pancreatic cancer: Pathogenesis and diagnosis. Asian Pac J Cancer Prev (2015) 16:5619–24. doi: 10.7314/APJCP.2015.16.14.5619 26320426

[3] Gupta R Amanam I Chung V. Current and future therapies for advanced pancreatic cancer. J Surg Oncol (2017) 116:25–34. doi: 10.1002/jso.24623 28591939

[4] Ilic M Ilic I. Epidemiology of pancreatic cancer. World J Gastroenterol (2016) 22:9694–705. doi: 10.3748/wjg.v22.i44.9694 PMC5124974 27956793

[5] Feins S Kong W Williams EF Milone MC Fraietta JA. An introduction to chimeric antigen receptor (CAR) T-cell immunotherapy for human cancer. Am J Hematol (2019) 94:S3–9. doi: 10.1002/ajh.25418 30680780

[6] Neelapu SS Locke FL Bartlett NL Lekakis LJ Miklos DB Jacobson CA. Axicabtagene ciloleucel CAR T-cell therapy in refractory Large b-cell lymphoma. N Engl J Med (2017) 377:2531–44. doi: 10.1056/NEJMoa1707447 PMC5882485 29226797

[7] Wang M Munoz J Goy A Locke FL Jacobson CA Hill BT. KTE-X19 CAR T-cell therapy in relapsed or refractory mantle-cell lymphoma. N Engl J Med (2020) 382:1331–42. doi: 10.1056/NEJMoa1914347 PMC7731441 32242358

[8] Cohen AD Garfall AL Stadtmauer EA Melenhorst JJ Lacey SF Lancaster E. B cell maturation antigen-specific CAR T cells are clinically active in multiple myeloma. J Clin Invest (2019) 129:2210–21. doi: 10.1172/JCI126397 PMC6546468 30896447

[9] Raje N Berdeja J Lin Y Siegel D Jagannath S Madduri D. Anti-BCMA CAR T-cell therapy bb2121 in relapsed or refractory multiple myeloma. N Engl J Med (2019) 380:1726–37. doi: 10.1056/NEJMoa1817226 PMC8202968 31042825

[10] Martinez M Moon EK. CAR T cells for solid tumors: New strategies for finding, infiltrating, and surviving in the tumor microenvironment. Front Immunol (2019) 10:128. doi: 10.3389/fimmu.2019.00128 30804938 PMC6370640

[11] Newick K O'Brien S Moon E Albelda SM. CAR T cell therapy for solid tumors. Annu Rev Med (2017) 68:139–52. doi: 10.1146/annurev-med-062315-120245 27860544

[12] Dai H Tong C Shi D Chen M Guo Y Chen D. Efficacy and biomarker analysis of CD133-directed CAR T cells in advanced hepatocellular carcinoma: a single-arm, open-label, phase II trial. Oncoimmunology (2020) 9:1846926. doi: 10.1080/2162402X.2020.1846926 33312759 PMC7714531

[13] Shi D Shi Y Kaseb AO Qi X Zhang Y Chi J. Chimeric antigen receptor-Glypican-3 T-cell therapy for advanced hepatocellular carcinoma: Results of phase I trials. Clin Cancer Res (2020) 26:3979–89. doi: 10.1158/1078-0432.CCR-19-3259 32371538

[14] Brown CE Badie B Barish ME Weng L Ostberg JR Chang WC. Bioactivity and safety of IL13Ralpha2-redirected chimeric antigen receptor CD8+ T cells in patients with recurrent glioblastoma. Clin Cancer Res (2015) 21:4062–72. doi: 10.1158/1078-0432.CCR-15-0428 PMC4632968 26059190

[15] O'Rourke DM Nasrallah MP Desai A Melenhorst JJ Mansfield K Morrissette JJD. A single dose of peripherally infused EGFRvIII-directed CAR T cells mediates antigen loss and induces adaptive resistance in patients with recurrent glioblastoma. Sci Transl Med (2017) 9:eaaa0984. doi: 10.1126/scitranslmed.aaa0984 28724573 PMC5762203

[16] Beatty GL O'Hara MH Lacey SF Torigian DA Nazimuddin F Chen F. Activity of mesothelin-specific chimeric antigen receptor T cells against pancreatic carcinoma metastases in a phase 1 trial. Gastroenterology (2018) 155:29–32. doi: 10.1053/j.gastro.2018.03.029 29567081 PMC6035088

[17] Rodriguez-Garcia A Palazon A Noguera-Ortega E Powell DJ Jr. Guedan S. CAR-T cells hit the tumor microenvironment: Strategies to overcome tumor escape. Front Immunol (2020) 11:1109. doi: 10.3389/fimmu.2020.01109 32625204 PMC7311654

[18] Kailayangiri S Altvater B Wiebel M Jamitzky S Rossig C. Overcoming heterogeneity of antigen expression for effective CAR T cell targeting of cancers. Cancers (Basel) (2020) 12:1075. doi: 10.3390/cancers12051075 PMC7281243 32357417

[19] Majzner RG Mackall CL. Tumor antigen escape from CAR T-cell therapy. Cancer Discov (2018) 8:1219–26. doi: 10.1158/2159-8290.CD-18-0442 30135176

[20] Poorebrahim M Melief J Pico de Coana Y LW S Cid-Arregui A Kiessling R. Counteracting CAR T cell dysfunction. Oncogene (2021) 40:421–35. doi: 10.1038/s41388-020-01501-x PMC7808935 33168929

[21] Ramakrishna S Barsan V Mackall C. Prospects and challenges for use of CAR T cell therapies in solid tumors. Expert Opin Biol Ther (2020) 20:503–16. doi: 10.1080/14712598.2020.1738378 32125191

[22] Saka D Gokalp M Piyade B Cevik NC Arik Sever E Unutmaz D. Mechanisms of T-cell exhaustion in pancreatic cancer. Cancers (Basel) (2020) 12(8). doi: 10.3390/cancers12082274 PMC7464444 32823814

[23] Hassan R Ho M. Mesothelin targeted cancer immunotherapy. Eur J Cancer (2008) 44:46–53. doi: 10.1016/j.ejca.2007.08.028 17945478 PMC2265108

[24] Le K Wang J Zhang T Guo Y Chang H Wang S. Overexpression of mesothelin in pancreatic ductal adenocarcinoma (PDAC). Int J Med Sci (2020) 17:422–7. doi: 10.7150/ijms.39012 PMC7053310 32174772

[25] Chou A Waddell N Cowley MJ Gill AJ Chang DK Patch AM. Clinical and molecular characterization of HER2 amplified-pancreatic cancer. Genome Med (2013) 5:78. doi: 10.1186/gm482 24004612 PMC3978667

[26] Feng K Liu Y Guo Y Qiu J Wu Z Dai H. Phase I study of chimeric antigen receptor modified T cells in treating HER2-positive advanced biliary tract cancers and pancreatic cancers. Protein Cell (2018) 9:838–47. doi: 10.1007/s13238-017-0440-4 PMC6160389 28710747

[27] Beatty GL Haas AR Maus MV Torigian DA Soulen MC Plesa G. Mesothelin-specific chimeric antigen receptor mRNA-engineered T cells induce anti-tumor activity in solid malignancies. Cancer Immunol Res (2014) 2:112–20. doi: 10.1158/2326-6066.CIR-13-0170 PMC3932715 24579088

[28] Adachi K Kano Y Nagai T Okuyama N Sakoda Y Tamada K. IL-7 and CCL19 expression in CAR-T cells improves immune cell infiltration and CAR-T cell survival in the tumor. Nat Biotechnol (2018) 36:346–51. doi: 10.1038/nbt.4086 29505028

[29] Forster R Davalos-Misslitz AC Rot A. CCR7 and its ligands: balancing immunity and tolerance. Nat Rev Immunol (2008) 8:362–71. doi: 10.1038/nri2297 18379575

[30] Yan Y Chen R Wang X Hu K Huang L Lu M. CCL19 and CCR7 expression, signaling pathways, and adjuvant functions in viral infection and prevention. Front Cell Dev Biol (2019) 7:212. doi: 10.3389/fcell.2019.00212 31632965 PMC6781769

[31] Mollica Poeta V Massara M Capucetti A Bonecchi R. Chemokines and chemokine receptors: New targets for cancer immunotherapy. Front Immunol (2019) 10:379. doi: 10.3389/fimmu.2019.00379 30894861 PMC6414456

[32] Tokunaga Y Sasaki T Goto S Adachi K Sakoda Y Tamada K. Enhanced anti-tumor responses of tumor antigen-specific TCR-T cells genetically engineered to produce IL-7 and CCL19. Mol Cancer Ther (2022) 21:138–48. doi: 10.1158/1535-7163.MCT-21-0400 34675119

[33] Adusumilli PS Cherkassky L Villena-Vargas J Colovos C Servais E Plotkin J. Regional delivery of mesothelin-targeted CAR T cell therapy generates potent and long-lasting CD4-dependent tumor immunity. Sci Transl Med (2014) 6:261ra151. doi: 10.1126/scitranslmed.3010162 PMC4373413 25378643

[34] Zhang L Kerkar SP Yu Z Zheng Z Yang S Restifo NP. Improving adoptive T cell therapy by targeting and controlling IL-12 expression to the tumor environment. Mol Ther (2011) 19:751–9. doi: 10.1038/mt.2010.313 PMC3070103 21285960

[35] Tanyi JL Stashwick C Plesa G Morgan MA Porter D Maus MV. Possible compartmental cytokine release syndrome in a patient with recurrent ovarian cancer after treatment with mesothelin-targeted CAR-T cells. J Immunother (2017) 40:104–7. doi: 10.1097/CJI.0000000000000160 28234665

[36] Fraietta JA Lacey SF Orlando EJ Pruteanu-Malinici I Gohil M Lundh S. Determinants of response and resistance to CD19 chimeric antigen receptor (CAR) T cell therapy of chronic lymphocytic leukemia. Nat Med (2018) 24:563–71. doi: 10.1038/s41591-018-0010-1 PMC6117613 29713085

[37] Vormittag P Gunn R Ghorashian S Veraitch FS. A guide to manufacturing CAR T cell therapies. Curr Opin Biotechnol (2018) 53:164–81. doi: 10.1016/j.copbio.2018.01.025 29462761

